# Research on the Carbon Credit Exchange Strategy for Scrap Vehicles Based on Evolutionary Game Theory

**DOI:** 10.3390/ijerph20032686

**Published:** 2023-02-02

**Authors:** Quan Wu, Wei Cheng, Zuoxiong Zheng, Guangjun Zhang, Haicheng Xiao, Chuan Wen

**Affiliations:** 1 Faculty of Transportation Engineering, Kunming University of Science and Technology, Kunming 650500, China; 2 Yunnan Engineering Survey and Design Institute Group Co., Ltd., Kunming 650500, China; 3 Faculty of Architecture, Civil and Transportation Engineering, Beijing University of Technology, Beijing 100124, China

**Keywords:** carbon credits, evolutionary game theory, scrap vehicles, transportation industry

## Abstract

In this article, we construct a game model that uses government regulators and scrap vehicle owners as the main parties to investigate the carbon credit exchange strategy of scrap vehicles using evolutionary game theory. The results were validated using Matlab simulation analysis to reveal the dynamic evolution process of the strategy of both sides of the game. A sensitivity analysis of the key parameters was conducted to explore the influence of each parameter on the evolution process and the stabilization trends. The study shows that (1) The time for the game system to reach a steady state is inversely related to the size of the initial willingness of the parties to cooperate. (2) In the mixed steady-state scenario, when the overall return differential between the positive and negative regulatory verification by government departments is positive, the steady state is participation and positive scrapping. (3) When the probability of the government verifying and being successful in verifying the punishment of the owner’s negative scrapping behavior increases, both parties of the game will eventually choose the strategy of participation and positive scrapping. When the cost of the government participation strategy and the cost of the government verification strategy increase, both sides of the game will eventually choose the strategy combination of no participation and positive scrapping. (4) When the owner’s reward for cooperating with the strategy, the owner’s cost of scrapping the vehicle, and the benefits of the owner’s negative cooperation strategy change, they will not change the strategy stability results but will affect the time it takes for the game system to reach a stable state. This study has theoretical implications for government policies in the scrapping industry and how to guide vehicle owners to actively scrap their vehicles.

## 1. Introduction

With the rapid socio-economic development in China, the national number of motor vehicles reached 406 million units at the end of June 2022 [1]. The continuous growth in motor vehicle ownership has introduced enormous pressure concerning urban traffic, which has aggravated traffic congestion and worsened environmental pollution in cities [2,3]. Due to the continuous progress in the automobile industry and the successive introduction of related management policies [4,5,6], the service life of vehicles is restricted. At the same time, there are still many problems in the scrap vehicle market, such as irregular procedures, poor management by management departments, the occupation of public resources by scrap vehicles and deliberate avoidance of scrapping by vehicle owners, leading to significant challenges in the scrap vehicle market.

At present, mitigating the carbon emissions in the field of scrap vehicles is challenged by substandard emission standards and poor energy utilization efficiency. Therefore, the development plans for scrap vehicle carbon emissions, the policy measures for scrap vehicles in the context of “low-carbon transportation”, and the contribution to the goal of net zero emissions in the transportation sector have become the current carbon emission research hotspots [7,8]. Carbon credits are carbon dioxide emission quotas established by the European Union, and companies or individuals earn “carbon coins” through low-carbon behavior that can be used in real-world scenarios to achieve zero carbon communities [2,9,10]. Carbon credits are currently implemented in Milan, Lisbon, Stockholm, and other cities, and have exhibited good results [11,12,13]. Carbon credits mainly promote a low-carbon life through government-led schemes and several domestic and foreign organizations, rewarding users who carry out low-carbon activities and providing guidance for popularizing the concept of low-carbon lifestyles. Based on the analysis of foreign experiences with carbon credit exploration and implementation, the carbon credits policy is applicable to the scrap vehicles market, proposed on the basis of the current situation in China’s transportation development [14,15,16,17], i.e., vehicle owners reaching the end of life limit are actively guided to complete vehicle scrapping to accumulate certain carbon credits for future low-carbon travel. However, how to determine the input costs, the benefits for the government and vehicle owners on both sides, and a stable state still require further research. In view of this, in order to further explore the feasibility of the carbon credit exchange strategy for scrap vehicles, the stability of the deal between the government and vehicle owners and the sensitivity of each influencing parameter are explored. In this article, we choose the government and vehicle owners as the main subjects and use evolutionary game theory to investigate the carbon credit exchange strategy for scrap vehicles. The results are verified using Matlab simulation analysis to reveal the dynamic evolution process of the strategies on both sides of the game, a sensitivity analysis of the key parameters is conducted, and then the influence of each parameter on the evolution process and the stability trend is explored. The study has theoretical guidance for government departments to formulate policies relating to the scrap vehicle industry and how to guide vehicle owners to actively scrap their vehicles.

## 2. Literature Review

In 1990, CARB, the U.S. Air Resources Board, took the lead in proposing the “Zero Emission Vehicle” CEV program, which mainly aimed to prevent pollutant emissions from motor vehicles [18,19]. With the gradual strengthening of the awareness of energy conservation and emission reduction, countries have proposed corresponding policy systems according to their own development, and the EU has set not only strict emission targets but also introduced a carbon credits system [16,20]. In China, a new energy vehicle credit management system, or NEV, has been proposed, which is a carbon credits policy based on the U.S. ZEV program, also called “double credit” [14,15,21,22]. The purpose is to curb vehicle carbon emissions through government measures that can benefit everyone.

Carbon credit exchange is an emission reduction measure wherein the government acts as the competent authority [23,24]; it mainly involves the interest relationship between the government and vehicle owners. Several scholars have studied the means to implement carbon credit exchange strategies [25,26,27,28], mainly using carbon emission forecasting models, low-carbon multi-objective planning models, and process model simulation methods. Wang et al. reviewed the measurement methods, analyses, and implementation policies of carbon credits in the transportation industry, describing the advantages and limitations of each piece of research and the strengths and limitations of each research method [29,30]. In terms of the factors influencing carbon credit exchange strategies, some scholars have analyzed the driving factors and development trends in transportation carbon emission [25,31,32,33], and Sun et al. used a three-dimensional gray correlation analysis model as a research method to demonstrate the time series gray correlation of each influencing factor with transportation carbon credits and regional gray correlation degrees [34]. In addition, the multivariate generalized Fisher exponential decomposition model [35], extended STIRPAT model [36,37], Kuznets curve model [38], and the GA-ELM hybrid heuristic algorithm [39] have been widely applied in the existing literature. Yuan et al. used the STIRPAT model combined with the scenario analysis method to explore carbon emissions, detailing the relationship between CO_2_ emissions and various influencing factors [40].

Evolutionary game theory is an important method for multiple parties to jointly coordinate optimization objectives [41], and is widely used in situations such as strategic positioning, business models, transportation carbon emissions, etc. Zhou et al. constructed a three-party evolutionary game model for wastewater companies, governments, and the public, and designed a reasonable reward and punishment mechanism [42]. Liu et al. applied the evolutionary game model to a two-level green supply chain consisting of green suppliers and green manufacturers, analyzed various internal and external factors affecting the behavior of both sides, and then numerically simulated the evolution and stabilization trends in coordinated emission reduction, with the results of the study identifying a variety of system evolution scenarios [43]. Recently, some scholars have also used game theory to study the carbon emission strategies between the government and automobile enterprises. Fang proposed two methods, GRNN and GRNN, to predict the number of scrap vehicles in China [44], and investigated the impact of government subsidies on scrap vehicle recycling [45]. In the low-carbon research of scrap vehicles, some scholars have analyzed the influencing factors of the development of the scrap vehicles recycling industry [46] and the cost of recycling end of life vehicles [47,48]. Zhang et al. studied the competition between illegal recycling and legal recycling groups in the scrap vehicle recycling market and established the evolutionary game model of the competition process between them [49].

Given the existing literature, carbon credit exchange is an important strategic measure, and multiple models have revealed its implementation potential. Scholars mostly study the influencing factors of strategies, and few studies have focused on the interest coordination relationship between the government and owners and the evolution trends in various other factors. Meanwhile, there are still many problems present in the scrap vehicle recycling market, and a reasonable and effective recycling system for scrap vehicles that actively guides vehicle owners to exchange a vehicle for carbon credits is an important means to solve a series of current problems, such as the overabundance of scrap vehicles, public resources occupied by scrap vehicles, and unregulated carbon credit trading.

In summary, this article investigates the carbon credit exchange strategy for scrap vehicles using evolutionary game theory, aiming to explore the evolutionary trends of the parameters of the carbon credit exchange process and the stability of the system.

## 3. Modeling

### 3.1. Model Assumptions

Both the government and the owners are rational actors pursuing certain interests, and the interests of both sides in cooperation will inevitably conflict, which is an important basis for the formation of the game relationship. Without changing the essence of the problem, some complex conditions are simplified. In order to show the game characteristics of the evolution of the cooperation between the two parties more intuitively, the modeling analysis is assumed to be carried out on the basis of satisfying the following scenario conditions.

**Hypothesis 1.**  *As the two rational subjects in this model, the government and the owner of a vehicle that has reached the end of life limit or standard will choose the most favorable solution for themselves, i.e., both wish to achieve the maximum economic benefits at the minimum economic cost, and both parties are in a state of incomplete information symmetry, optimizing and correcting their strategies to reach equilibrium through repeated games.*

**Hypothesis 2.**  *The government can choose to participate in the carbon credit exchange strategy for scrap vehicles to provide incentives for the carbon credit exchange strategy for scrap vehicles and supervise and manage the effective implementation of the strategy, or it can choose not to participate. Let the probability of participation be x (0 ≤ x ≤ 1), then the probability of non-participation is 1 − x. Vehicle owners can choose to actively scrap their vehicles in exchange for certain carbon credits to facilitate future low-carbon travel and personal carbon trading, or they can choose to negatively scrap their vehicles and continue to use them illegally until they are investigated and punished by the management. Let the probability of actively scrapping their vehicles be y (0 ≤ y ≤ 1), then the probability of negatively scrapping their vehicles is 1 − y.*

**Hypothesis 3.**  *The government can provide certain carbon credit incentives based on the proactive scrapping of vehicles and provide certain low-carbon travel restrictions and other penalties to vehicle owners who meet the scrapping standards but do not scrap their vehicles.*

**Hypothesis 4.**  *The government’s low-carbon travel restrictions and other penalties for vehicle owners are predicated on participation in the carbon credit exchange strategy for scrap vehicles and that the owners do not dispose of their vehicles through abandonment, in accordance with the relevant requirements.*

### 3.2. Parameter Assumptions and Model Construction

The government and the owner, as the stakeholders of this game analysis, both expect to maximize their own interests by adjusting their strategies, and there is a dynamic game relationship between them. In order to facilitate understanding and model building, the model parameters are assigned and organized as follows in Table 1.

Based on the above assumptions and the strategic choices of different subjects, the game payoff matrix can be constructed for different scenarios [49], as shown in Table 2.

Benefits for vehicle owners actively scrapping their vehicles:

(1) $$U_{y}=x(Q-s+u)+(1-x)(Q-s).$$

Benefits for the owners of negatively scrap vehicles:

(2) $$U_{\left(1-y\right)}=x\left[Q+p-v(s+t)\right]+\left(1-x\right)\left(Q+p\right).$$

Average expected return for vehicle owners:

(3) $$\begin{array}{cc} {\bar{U}}_{1} & =yU_{y}+\left(1-y\right)U_{\left(1-y\right)}\\ & =y\left[x(Q-s+u)+(1-x)(Q-s)\right]+\left(1-y\right)\left\{x\left[Q+p-v(s+t)\right]+\left(1-x\right)\left(Q+p\right)\right\}\\ & =xy\left(u+vs-vt\right)+x\left(vt-vs\right)-y\left(s+p\right)+Q+p. \end{array}$$

Benefits of government participation in carbon credit exchange strategies:

(4) $$U_{x}=y\left(R-r-u-w\right)+\left(1-y\right)\left(vt-r-w\right).$$

Benefits of government non-participation in carbon credit exchange strategies:

(5) $$U_{\left(1-x\right)}=yR$$

Average expected return for the government:

(6) $$\begin{array}{cc} {\bar{U}}_{2} & =xU_{x}+\left(1-x\right)U_{\left(1-x\right)}\\ & =x\left[y\left(R-r-u-w\right)+\left(1-y\right)\left(vt-r-w\right)\right]+\left(1-x\right)yR\\ & =x\left(vt-r-w\right)+yR-xy\left(u+vt\right). \end{array}$$

Behavioral evolution of both sides of the game replicates the dynamic equation:

(7) $$F(x,y)=\frac{d_{x}}{d_{t}}=x(1-x)[U_{x}-U_{\left(1-x\right)}]=x(1-x)[\left(1-y\right)vt-yu-r-w]$$

(8) $$G(x,y)=\frac{d_{y}}{d_{t}}=y(1-y)[U_{y}-U_{\left(1-y\right)}]=y(1-y)[x(u+vt+vs)-s-p].$$

## 4. Analysis

Let $F(x,y)=\frac{d_{x}}{d_{t}}=0$ and $G(x,y)=\frac{d_{y}}{d_{t}}=0$ to obtain five equilibria [50]:

Respectively, $\{\left(0,0\right),\left(0,1\right),\left(1,0\right),\left(1,1\right),\left(\frac{s+p}{vt+vs+u},\frac{vt-r-w}{vt+u}\right)\}$.

Order $A=\frac{s+p}{vt+vs+u},B=\frac{vt-r-w}{vt+u}$.

### 4.1. Owner Stability Analysis

The derivation of the owner replication dynamic equation yields:

(9) $$\frac{d_{F\left(x,y\right)}}{d_{x}}=\frac{\partial \left[x(1-x)\right]}{\partial x}\left[\left(1-y\right)vt-yu-r-w\right]=(1-2x)\left[\left(1-y\right)vt-yu-r-w\right],$$

So, the inference is as follows:

(1) Let (1−y)vt−yu−r−w=0, i.e., $y=\frac{vt-r-w}{1+vt}$, then the results show that F(x,y)=0, at which point the probability of scrapping the vehicle, whether actively or not, reaches a stable point for the owner.

(2) Let (1−y)vt−yu−r−w≠0, i.e., $y\neq \frac{vt-r-w}{1+vt}$, let F(x,y)=0, i.e., x(1−x)[(1−y)vt−yu−r−w]=0, and solve the equation to obtain x=0 and x=1. If $y>\frac{vt-r-w}{1+vt}$, we can obtain $\frac{d_{F\left(x,y\right)}}{d_{x}}|x=0>0$ and $\frac{d_{F\left(x,y\right)}}{d_{x}}|x=1>0$, so x=1 is the stable strategy. At this time, the vehicle owner will actively cooperate with the carbon credit exchange strategy for scrapping the vehicle in order to receive the reward from the carbon credit exchange strategy provided by the government and avoid unnecessary penalties and losses and finish the scrapping work of the vehicle. On the contrary, x=0 is an unstable strategy; the vehicle owner will take a chance and not cooperate with the government’s vehicle scrapping policy when the vehicle reaches the scrapping condition and will continue to use the scrap vehicle [49].

### 4.2. Government Stability Analysis

The derivation of the government replication dynamic equation yields:

(10) $$\frac{d_{G\left(x,y\right)}}{d_{y}}=\frac{\partial \left[y(1-y)\right]}{\partial y}\left[x\left(u+vt+vs\right)-s-p\right]=\left(1-2y\right)\left[x\left(u+vt+vs\right)-s-p\right]$$

Thus,

(1) Let x(u+vt+vs)−s−p=0, i.e., $x=\frac{s+p}{u+vt+vs}$, then the results show that G(x,y)=0, which is a stable point for whether the government participates in the abandoned vehicle carbon credit exchange strategy or not.

(2) Let x(u+vt+vs)−s−p≠0, i.e., $x\neq \frac{s+p}{u+vt+vs}$, let G(x,y)=0, i.e., y(1−y)[x(u+vt+vs)−s−p]=0, then solve the equation to obtain y=0 and y=1. If $x>\frac{s+p}{u+vt+vs}$, we can obtain $\frac{d_{G\left(x,y\right)}}{d_{x}}|y=0>0$ and $\frac{d_{G\left(x,y\right)}}{d_{x}}|y=1>0$, so y=0 is the unstable strategy. At this time, the government department hopes to save the costs of participating in the carbon credit exchange strategy and the incentive expenses for vehicle owners, hoping that owners can take the initiative to scrap vehicles that reach the abandoned condition and consciously reduce carbon emissions in order to enjoy the dividends of the “carbon peaking and carbon neutrality” strategy provided through the scrapping of vehicles. In this case, the government will choose not to participate in the carbon credit exchange strategy for scrap vehicles. On the contrary, y=1 is a stable strategy, i.e., the government departments hope to control the carbon emissions due to scrap vehicles through certain strategies and actively participate in the carbon credit exchange strategy for scrap vehicles, and at the same time provide incentives and encouragement to vehicle owners who actively cooperate with the strategy so as to balance the implementation of the carbon credit exchange strategy for scrap vehicles through economic leverage, and finally achieve the purpose of regulating vehicle owners, energy savings, and emission reductions, and also impose corresponding penalties on owners who violate the law. At the same time, they will also impose penalties on the owners of non-compliant vehicles so that they can obtain penalty revenue.

### 4.3. Mixing Stability Analysis

According to the theorem of nonlinear differential equations, differential equations can be used to analyze the dynamic change process of the game system, and the stability degree of the system equilibrium point can be judged using the Jacobi matrix [51]. The system of replicated dynamic equations constructed by Equations (7) and (8) can be calculated to obtain the Jacobi matrix and the trace expression as follows [52]:

(11) $$J=\left[\begin{array}{l} \frac{d_{F\left(x,y\right)}}{d_{x}}\frac{d_{F\left(x,y\right)}}{d_{y}}\\ \frac{d_{G\left(x,y\right)}}{d_{x}}\frac{d_{G\left(x,y\right)}}{d_{y}} \end{array}\right]=\left[\begin{array}{l} (1-2x)\left[(1-y)vt-yu-r-w\right]\cdots \cdots x\left(x-1\right)\left(u+vt\right)\\ y\left(1-y\right)\left(u+vt+vs\right)\cdots \cdots \cdots \cdots \cdots \cdots \left(1-2y\right)\left[x\left(u+vt+vs\right)-s-p\right] \end{array}\right]$$

The determinant of J is as follows:

(12) $$\begin{array}{l} DetJ=(1-2x)\left[(1-y)vt-yu-r-w\right]\left(1-2y\right)\left[x\left(u+vt+vs\right)-s-p\right]\\ -x\left(x-1\right)\left(u+vt\right)\left(1-2y\right)\left[x\left(u+vt+vs\right)-s-p\right] \end{array}$$

The trace of J is as follows:

(13) TrJ=(1−2x)[(1−y)vt−yu−r−w]+(1−2y)[x(u+vt+vs)−s−p].

When DetJ>0 and TrJ<0 are local asymptotically stable immobile points, i.e., the evolutionary stabilization strategy (ESS) of the system [53], at this time, the values of the local equilibrium points in this evolutionary game system can be obtained according to the analysis of the positive and negative values of the determinants and traces. As shown in Table 3.

According to the above analysis, it can be seen that there are 16 scenarios for the evolutionary process, but because the range of values of a and d is always negative, it is known that there are only four evolutionary processes that actually satisfy the game system. Next, we discuss the stability of the strategy of the evolutionary game between the government and the owner under different constraints and analyze the stability of the evolutionary process in all cases; however, we will not analyze the cases where a>0 or d>0 [54].

Scenario 1: For a<0,b<0,c<0,d<0, the stabilities of the equilibrium points of the game system are shown in Table 4. The evolutionary stabilization strategy (ESS) of the game system, in this case, is $E_{1}\left(0,0\right)$, where the saddle points are (0,1) and (1,0), and the unstable point is (1,1).

Specifically, if the government is unwilling to participate in the carbon credit exchange strategy for scrap vehicles, and vehicle owners are unwilling to actively scrap their vehicles, the benefits that vehicle owners continue to enjoy by negatively scrapping their vehicles are less than the costs required to scrap their vehicles, and vehicle owners will bear the risk of positive government verification, and thus be forced to scrap their vehicles. Regardless of whether the owner chooses to actively scrap their vehicle, the overall revenue and expenditure of the government are always negative; therefore, after numerous games, it will tend toward negative regulation. The advantage of such a game is that, for the government, it can effectively reduce financial expenditure. Furthermore, in relation to the vehicle owners, because they can continue to enjoy the use of the vehicle and economic and daily expenditure savings, they will tend to violate the use of vehicles for more benefits. However, for the long-term strategy of transportation carbon emissions, this move is not conducive to the effective implementation of the “double carbon” strategy, and environmental pollution and transportation carbon emissions cannot be effectively curbed and should not be promoted.

Scenario 2: For a<0,b>0,c<0,d<0, the stabilities of the equilibrium points of the game system are shown in Table 5. The evolutionary stabilization strategy (ESS) of the game system in this case is $E_{3}\left(1,0\right)$, where the saddle points are (0,0) and (0,1), and the unstable point is (1,1).

Specifically, the government department prefers to participate in the carbon credit exchange strategy for scrap vehicles. The intervention of the government department leads owners to actively scrap their vehicles in exchange for certain carbon credits, and the government will provide certain incentives to vehicle owners who actively scrap their vehicles, and the overall gain difference between positive and negative regulation and verification by the government department is positive and will tend toward positive regulation after numerous games. At the same time, since the verification measures will investigate and deal with some behaviors that reach the abandoned limit but do not scrap the vehicle, the behavior will be punished by the government, and the government will gain some revenue from this. Furthermore, if the penalty revenue is greater than the cost of verification, the government is more inclined to carry out strict supervision and verification, which will increase the revenue and ensure the effective implementation of the carbon credit exchange strategy for scrap vehicles. This will introduce more benefits to low-carbon transportation and further promote the fulfillment of China’s peak carbon and carbon neutrality goals [55].

Scenario 3: For a<0,b<0,c>0,d<0, the stabilities of the equilibrium points of the game system are shown in Table 6. The evolutionary stabilization strategy (ESS) of the game system in this case is $E_{1}\left(0,0\right)$, where the saddle points are (0,1) and (1,1), and the unstable point is (1,0).

Specifically, regardless of whether vehicle owners choose to actively scrap their vehicles or negatively scrap their vehicles, the benefit difference between the government’s adoption of positive regulation and verification and the remaining cost expenditure is always negative; therefore, it will tend not to participate in the carbon credit exchange strategy for scrap vehicles, while negatively regulating and verifying. As a result of the negative regulation and verification of government departments, owners actively scrapping their vehicles do not receive incentives from local governments, and the scrapping of vehicles still requires certain costs, so the overall benefit difference between owners undertaking active scrapping and negative scrapping is negative, and the overall benefit of negative scrapping is greater than the expenses and penalties they receive, which will tend toward negative scrapping.

Scenario 4: For a<0,b>0,c>0,d<0, the stabilities of the equilibrium points of the game system are shown in Table 7. In this case, the determinants of the Jacobi matrix are all negative, so there is no ESS in the evolutionary game model, and the two sides of the game will move around the center of the spiral, showing: “Owner violates the negative scrapping of vehicle—Government active supervision and verification—Owner actively scrapping vehicles—Government negative supervision and verification—Owner’s violation and negative scrapping”.

Specifically, when the whole system tends towards balance and stability, the government will not actively participate in the carbon credit exchange strategy for scrap vehicles, attempting to maintain the balance through the macro-regulation of the market, reducing unnecessary financial expenses and saving the manpower and resources needed for the verification strategy. However, vehicle owners, if there are no government interventions, will try to maximize their own interests, leading to the trend of negative vehicle scrapping, which will hinder the effective implementation of the carbon neutrality strategy, which is not conducive to the original purpose of environmental protection. A government regulator, in order to effectively curb the occupation of public resources due to scrap vehicles and reduce the carbon emissions and environmental pollution originating from scrap vehicles as much as possible, will implement the carbon credit exchange strategy for scrapping vehicles and increase supervision and verification efforts so as to achieve the goal. After the government department implements the strategy and increases verification efforts, vehicle owners will actively participate in the strategy and cooperate with the “carbon peaking and carbon neutrality“ goals. With the evolution of time, the government department will be negative and provide the vehicle owners with an opportunity to take advantage of the strategy; thus, the strategy will not be effectively implemented, the evolution of the game between the government and vehicle owners will show a cyclic pattern, and the equilibrium point of the evolution process will not be found. The central point is (A,B), and the dynamic adjustment of various parameters in the evolution process can be made. The center point will also exhibit dynamic changes.

## 5. Results and Discussion

### 5.1. Simulation Analysis of Stability Results in Different Scenarios

**Figure 1:**
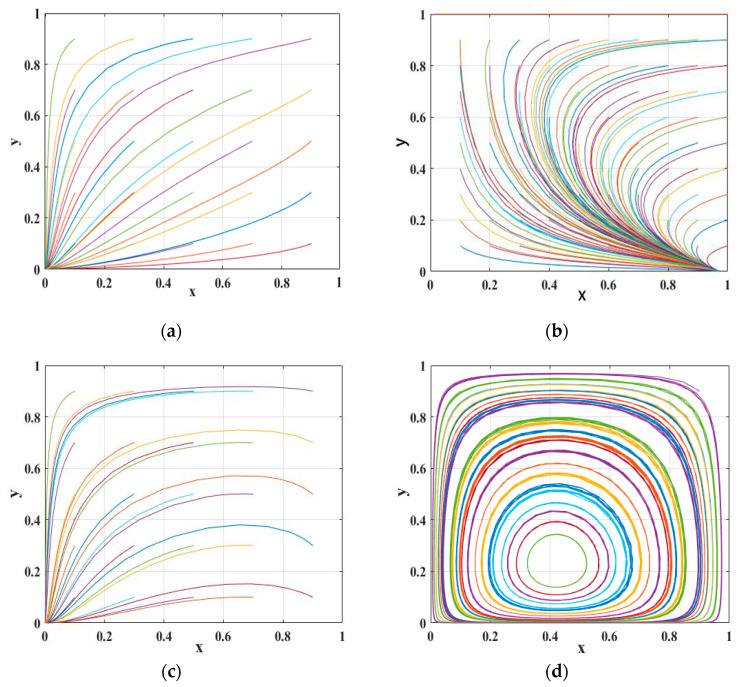
Simulation results of four scenarios. (**a**) Scenario 1; (**b**) Scenario 2; (**c**) Scenario 3; (**d**) Scenario 4.

Based on the above theoretical analysis, the evolutionary behavior process of the game between the government and vehicle owners is verified and analyzed numerically by using Matlab with the help of constraints and replicated dynamic equations. The relevant parameters are set in the following, and the simulation results can be found to be consistent with the above analysis. The simulation results are shown in Figure 1.

Scenario 1: The relevant parameters are taken as *t* = 8; *v* = 0.2; *u* = 4; *r* = 2; *w* = 2; *s* = 6; and *p* = 6. The simulation results are shown in Figure 1a. It can be seen that with the increase in the number of iterations of the evolutionary game, the proportion of both sides of the game gradually choosing the non-participation strategy and negative scrapping strategy combination increases.

Scenario 2: The relevant parameters are taken as *t* = 6; *v* = 0.8; *u* = 2; *r* = 2; *w* = 2; *s* = 6; and *p* = 6. The simulation results are shown in Figure 1b. With the increase in the number of iterations of the evolutionary game, the government tends to participate in the carbon credit exchange strategy for scrap vehicles, and the proportion of owners negatively scrapping their vehicles increases gradually, and the final steady state is participation strategy and active scrapping.

Scenario 3: The relevant parameters are taken as *t* = 8; *v* = 0.8; *u* = 4; *r* = 2; *w* = 2; *s* = 6; and *p* = 6. The simulation results are shown in Figure 1c, and it is easy to find that the benefits obtained by both sides of the game are negative, and both sides tend to choose the strategy combination of non-participation strategy and negative scrapping. This behavior affects the carbon credit transportation strategy.

Scenario 4: The relevant parameters are taken as *t* = 18; *v* = 0.8; *u* = 20; *r* = 2; *w* = 2; *s* = 6; and *p* = 6. The simulation results are shown in Figure 1d. At this time, the system has no ESS, and the combination of strategies chosen by both sides of the game will have a circular motion around the center point.

### 5.2. Both Participating Subjects Initial Willingness Sensitivity Analysis

From the above analysis and numerical simulation results, it can be seen that only the evolutionary game system of both government and vehicle owners under the conditions of scenarios 2 and 4 will reach the optimal strategy combination, i.e., participation strategy and active scrapping. However, the optimal strategy combination in scenario 4 is a cyclic process with instability and will face more complex situations in the implementation process, which is difficult to achieve in practical situations and not very operative. Therefore, under the conditions of scenario 2, how to increase the probability of the optimal strategy combination becomes a problem that needs to be solved.

**Figure 2:**
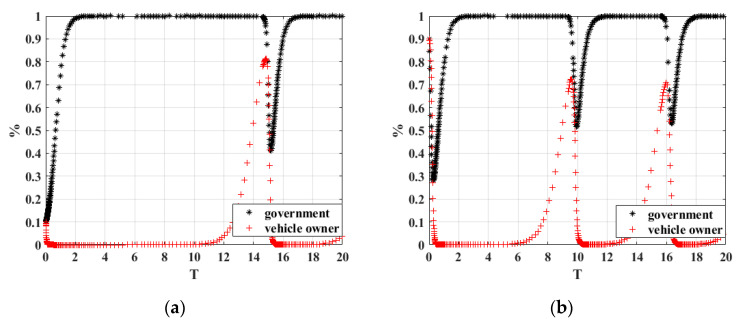
Simulation results with different initial probabilities. (**a**) Initial probability of (0.1, 0.1); (**b**) Initial probability of (0.9, 0.9).

Firstly, we analyzed the influence of the game parties on the evolutionary results under different initial willingness conditions. The initial probabilities of the two sides of the game are (0.1, 0.1) and (0.9, 0.9), respectively, and the parameters are taken with reference to scenario 2. The simulation results are shown in Figure 2. It can be found that when the government chooses the “participation strategy” and the owner chooses “active scrapping” with the probability of 0.1, with the change in evolution time, the system initially evolves the stable strategy combination of participation strategy and active scrapping when the time point reaches 14. When the probability of the initial strategy choice of both sides of the game is raised to 0.9, the time interval for the sudden change in the system strategy combination becomes shorter, and the overall trend in the evolutionary stable strategy combination is participation strategy and active scrapping.

According to the above analysis, the initial willingness will affect the evolutionary results, and in order to transform the final evolutionary stable strategy combination into the ideal strategy combination, it is necessary to take corresponding measures to improve the government’s enthusiasm to participate in the carbon credit exchange strategy and active verification strategy for scrap vehicles, and improve the possibility of vehicle owners to actively scrap their vehicles. This will be important in reducing the carbon emissions from scrap vehicles and eliminating the use of public resources.

### 5.3. Sensitivity Analysis of the Main Parameters of the System

In order to increase the probability of the ideal strategy combination and ensure the successful achievement of the “double carbon” goal, it is also possible to change the values of the main parameters of the system. Next, a sensitivity analysis of each parameter in the game system was conducted to discover the influence of each parameter on the system. The initial probabilities of the two sides of the game are assumed to be (0.5, 0.5) under the numerical simulation conditions of scenario 2, and the values of the other parameters are kept constant while changing the values of the analyzed parameters.

**Figure 3:**
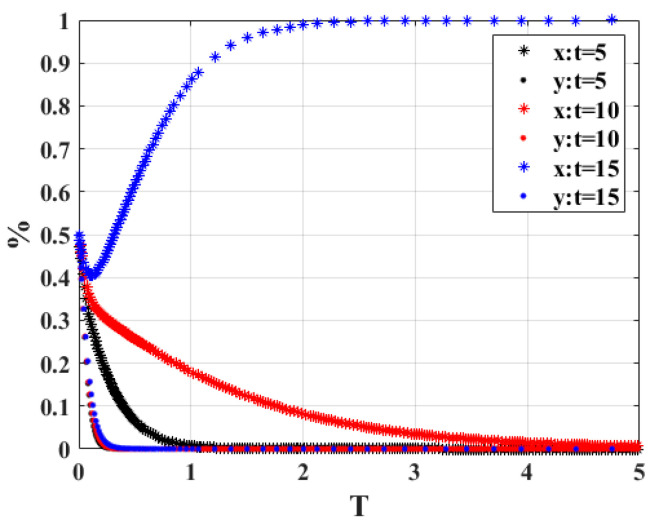
Sensitivity analysis of “*t*”.

**Figure 4:**
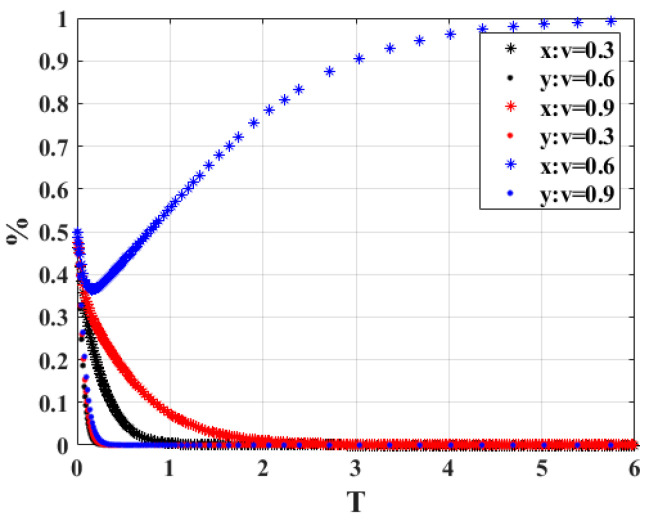
Sensitivity analysis of “*v*”.

**Figure 5:**
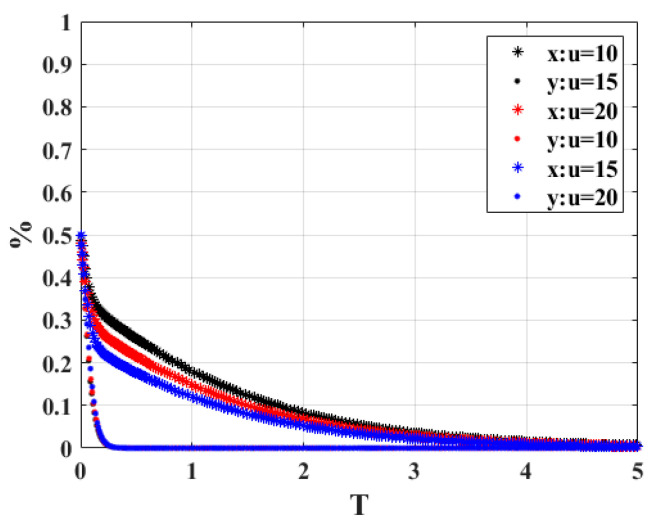
Sensitivity analysis of “*u*”.

**Figure 6:**
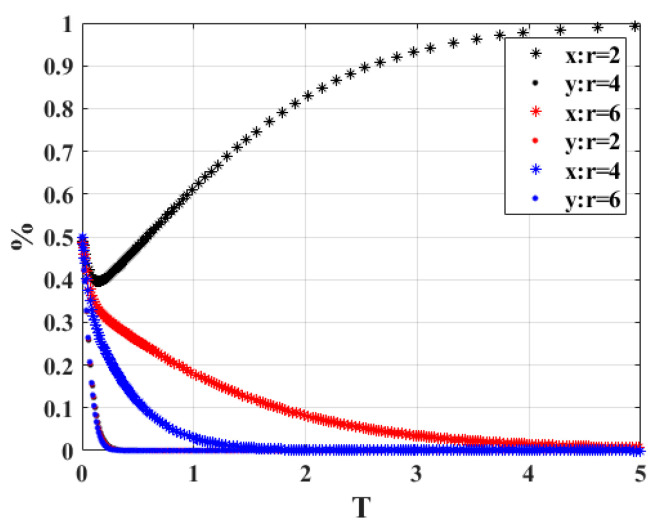
Sensitivity analysis of “*r*”.

**Figure 7:**
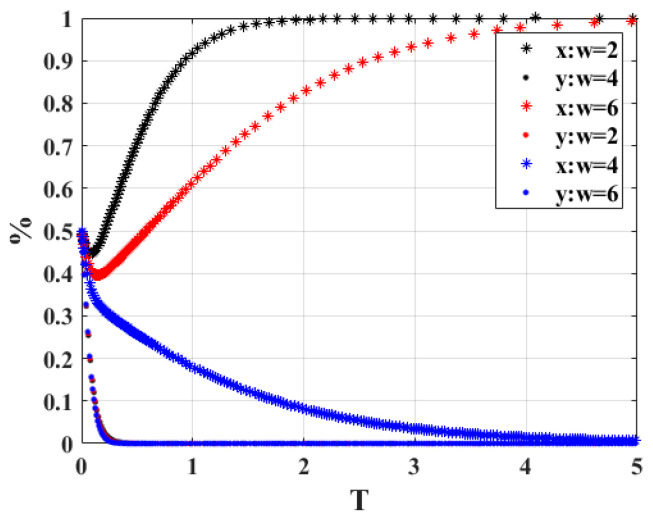
Sensitivity analysis of “*w*”.

**Figure 8:**
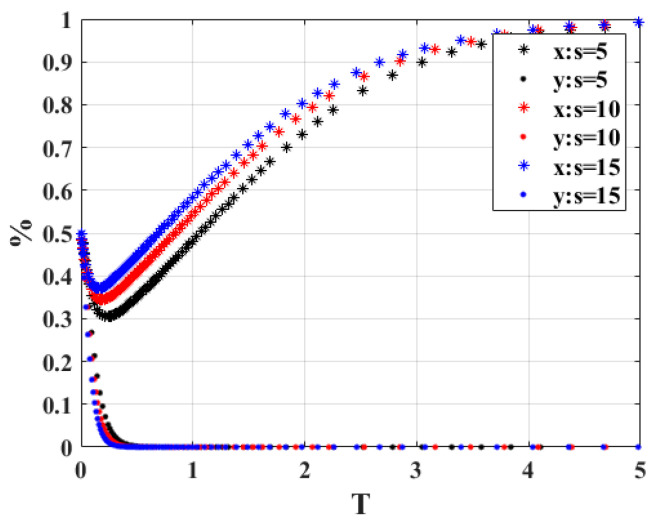
Sensitivity analysis of “*s*”.

**Figure 9:**
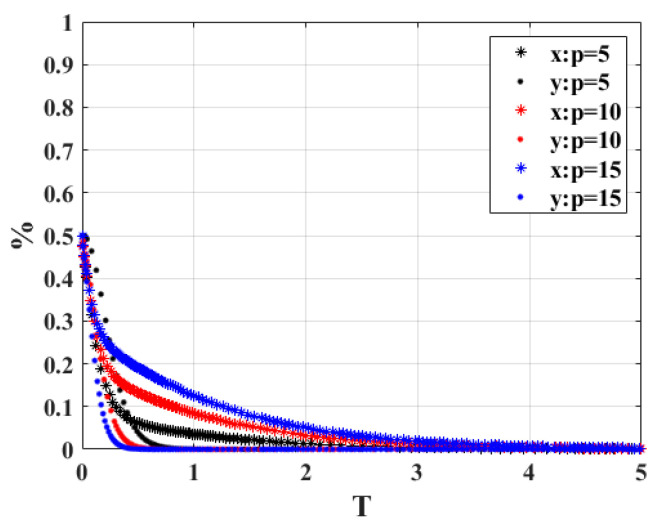
Sensitivity analysis of “*p*”.

1. When the government verifies that the penalty for the owner’s behavior of not actively scrapping the vehicle “*t*” is 5, 10, and 15, respectively, the strategy choices of both sides of the system game are shown in Figure 3. When *t* = 5 and *t* = 10, the government and vehicle owners will eventually choose the strategy combination of non-participation strategy and active scrapping, but when *t* increases to 15, as time passes, the game will eventually choose the strategy combination of participation strategy and active scrapping. In terms of the equilibrium time point, it can be seen that the time required for the owner to reach the steady state is much less than that required by the government, and as the value of *t* increases, more time is required for the government to reach the equilibrium state. The results indicate that when the penalty for car owners increases, the government prefers the proactive engagement strategy for active regulation, and it can achieve more profit from fines. At the same time, owners actively cooperate with the strategy to complete the scrapping of scrap vehicles, which will reduce carbon emissions.

2. When the probability of vehicle owners not actively scrapping their vehicles is verified by the government, “*v*” is 0.3, 0.6 and 0.9, respectively, the strategy choices of both sides of the system game are shown in Figure 4. When *v* = 0.3 and *t* = 0.6, the strategy combination of government and vehicle owners will be non-participation strategy and active scrapping, but when *v* increases to 0.9, the strategy combination of participation strategy and active scrapping will be chosen by both sides of the game as time passes. Additionally, it is not difficult to find that the government supervisor takes more time to reach the equilibrium state in the system. The results show that when the probability of the vehicle owner’s behavior of not actively scrapping the vehicle is verified by the government increases, the government department tends to participate in the strategy while actively verifying the vehicle owner who does not cooperate with the strategy, so that the vehicle owner actively cooperates with the government department and actively scraps the vehicle, resulting in the evolutionary system tending to equilibrium.

3. When the owner cooperates with the strategic behavior and the corresponding rewards “*u*” are 10, 15, and 20, respectively, the strategy choices of both sides of the system game are shown in Figure 5. It is easy to find that regardless of the value of *u*, both sides of the game will eventually choose the strategy combination of non-participation strategy and active scrapping. However, as the reward increases, the time required to reach a stable strategy for governmental behavior is also reduced. This indicates that the size of the incentive has an impact on the time to reach the stable state of the system, and the owners can be made to actively scrap their vehicles by improving other conditions in different time periods. Therefore, adjusting the appropriate size of the reward is also necessary for the stability of the gaming system.

4. When the cost “*r*” of the government’s participation in the carbon integral exchange strategy of scrap vehicles is 2, 4, and 6, respectively, the strategy choices of both parties in the system game are shown in Figure 6. According to the figure, when *r* = 2, the stability of the strategy combination of the two sides of the game is finally maintained at participation strategy and active scrapping, and as the value of *r* increases, i.e., when *r* = 4 and *r* = 6, the two sides of the game will finally choose the strategy combination of non-participation strategy and active scrapping, and as the cost of government participation in the strategy is higher, the time required to evolve to the stable point state is shorter. It is easy to understand that if the cost of the government involvement strategy is low, on the one hand, it can deter vehicle owners from actively scrapping their vehicles, and on the other hand, it can save financial expenditure, and the government is willing to actively participate in the strategy because the overall benefit of the system is positive. As the cost of participation increases, when the benefit obtained is lower than the high cost of participation, the government departments tend toward the non-participation strategy, and the game time is shortened as the cost increases.

5. The cost required for the government department to verify the strategy when “*w*” is 2, 4, and 6, respectively, and the strategy choices of both sides of the game are shown in Figure 7. According to the analysis results, when *w* = 2 and *w* = 4, the strategy combination of both sides of the game finally remains at participation strategy and active scrapping, and when *w* = 6, the government department and the car owner will choose the non-participation strategy and active scrapping game combination. As can be seen in the figure, when *w* is taken as either too small or too large, it will shorten the time for the system to reach the equilibrium stability point. The value of *w* value has little effect on the owners to reach the stability state; both tend toward active scrapping. The cost of the verification strategy is also correlated with the penalty obtained, and the government will be reluctant to participate in the strategy if the cost of verification is too high and the benefits of the penalties obtained are too low to cover the costs. Therefore, the cost of the appropriate verification strategy will directly affect how the gaming system is chosen.

6. The strategy choice of both sides of the system game is shown in Figure 8, when the cost of the owner’s scrap vehicle “*s*” is 5, 10 and 15, respectively. According to the analysis results, it can be seen that regardless of the value of *s*, the strategy combination between the government and the vehicle owner game will eventually stabilize at participation strategy and active scrapping. In terms of the time required to reach stability, the time for both sides of the game to reach stability decreases as the cost of scrapping vehicles increases. The cost of scrapping a vehicle does not affect the outcome of the game, but the necessary macroeconomic regulation to ensure a stable market equilibrium is also necessary.

7. The strategy choice of both sides of the system game is shown in Figure 9 when the owner does not cooperate with the strategy and scraps the vehicle negatively while receiving the benefits “*p*” of 5, 10, and 15, respectively. According to the analysis results, it can be seen that regardless of the value of *p*, the strategy combination of the two sides of the game will eventually stabilize at non-participation strategy and active scrapping. However, as the value of *p* increases, the time for the government to reach stability will be extended, showing a positive correlation and the time for the owners to reach stability will be shortened, showing a negative correlation. This indicates that the strategy finally chosen by the government department will not change because of the benefits received by the owners for negatively scrapping their vehicles but can regulate the time for the system to reach stability by the benefits received. Due to the imperfection of relevant laws and regulations, the advantages and disadvantages of negative vehicle scrapping cannot be clearly identified yet, so the next step is for government departments to continue to improve relevant laws and regulations, improve the operability of the interests of both parties in the game system, improve the reward and punishment system and carbon credit exchange strategy, force vehicle owners to scrap their vehicles actively, and carry out the carbon credit exchange strategy for scrap vehicles.

## 6. Conclusions

This article systematically investigates the possibility of whether the government and vehicle owners adopt a combination of carbon credit exchange strategies for scrap vehicles through a two-sided game model, and based on simulation design validation and sensitivity analysis, the following specific conclusions are obtained.

First, the time for the game system to reach the steady state is inversely related to the size of the initial willingness of both parties to cooperate; when the probability of initial willingness to cooperate increases, the time for the game system to reach the steady state will be shortened.

Second, in the mixed steady-state scenario, only when the overall benefit difference between positive and negative regulatory verification by government departments is positive, does the government tend to participate in the carbon credit exchange strategy for scrap vehicles. As the number of iterations of the evolutionary game increases and the proportion of vehicle owners actively scrapping their vehicles gradually increases, the final steady state is participation strategy and active scrapping. The remaining cases all lead to negative vehicle scrapping or no stable point for vehicle owners.

Third, the strategy choice of the government and vehicle owners depends on the size of the initial willingness of both parties to cooperate and the size of various influencing parameters. When the government verifies that the owner’s negative scrapping behavior is punished and the probability of being verified increases, both sides of the game will eventually choose the strategy combination of participation and active scrapping; when the cost of the government participation strategy and the cost required by government departments to verify the strategy increases, both sides of the game will eventually choose the strategy combination of non-participation and active scrapping.

Fourth, when the reward received by the owner for cooperating with the policy, the cost of scrapping the vehicle by the owner, and the benefit owned by the owner for negatively cooperating with the policy are changed, they will not change the policy stability results, but it will affect the time required for the system to reach the steady state.

Carbon credits are a persistent government strategy involving multiple participants. In this paper, only the game relationship between the government and vehicle owners was studied, and the interests of market traders were not considered, which has some limitations. The stable state between the government, vehicle owners, and the end of life market can be studied in the future to yield more applicable conclusions.

## Figures and Tables

**Table 1:** Model parameter assignment table.

Parameter Assignment	Parameter Meaning	Value Range
*x*	Probability that the government will choose an engagement strategy.	0 ≤ *x* ≤ 1
*y*	Probability of the owners choosing to scrap their vehicles.	0 ≤ *y* ≤ 1
*r*	Costs of government participation in scrap vehicles carbon credit exchange strategies.	*r* > 0
*Q*	Normal earnings for owners.	*Q* > 0
*s*	Costs for the owners to scrap their vehicles.	*s* > 0
*R*	Positive economic benefits to the “carbon peaking and carbon neutrality” strategy through the cooperation of vehicle owners with the strategy.	*R* > 0
*p*	The owner does not cooperate with the strategy and benefits from the negative scrapping of the vehicle.	*p* > 0
*t*	Government verification found that the owner of the vehicle participated in negative scrapping behavior and received a penalty.	*t* > 0
*u*	The owner cooperates with the strategic behavior and the corresponding reward is obtained.	*u* > 0
*v*	The probability that the negative scrapping behavior of vehicle owners will be verified by the government.	0 ≤ *v* ≤ 1
*w*	Cost of verification strategy for government departments.	*w >* 0

**Table 2:** The evolutionary game payoff matrix of both parties.

Gaming Subjects		Government Strategy	
		Participation *x*	Non-Participation (1 − *x*)
Ownership Strategies	Active scrapping, *y*	Q−s+u;R−r−u−w	Q−s;R
	Negative scrapping (1 − *y*)	Q+p−v(s+t);vt−r−w	Q+p;0

**Table 3:** Characteristics of local equilibrium point taking values.

Balance Point	Eigenvalue	Determinant	Trace
$E_{1}\left(0,0\right)$	$\lambda_{11}=a$, $\lambda_{12}=b$	$\lambda_{11}\cdot \lambda_{12}$	$\lambda_{11}+\lambda_{12}$
$E_{2}\left(0,1\right)$	$\lambda_{21}=-a$, $\lambda_{22}=d$	$\lambda_{21}\cdot \lambda_{22}$	$\lambda_{21}+\lambda_{22}$
$E_{3}\left(1,0\right)$	$\lambda_{31}=c$, $\lambda_{32}=-b$	$\lambda_{31}\cdot \lambda_{32}$	$\lambda_{31}+\lambda_{32}$
$E_{4}\left(1,1\right)$	$\lambda_{41}=-c$, $\lambda_{42}=-d$	$\lambda_{41}\cdot \lambda_{42}$	$\lambda_{41}+\lambda_{42}$
$E_{5}\left(A,B\right)$	——	——	——

Notes: a=−p−s,b=−r+vt−w,c=−p−s+u+sv+vt,d=−r−u−w

**Table 4:** Scenario 1 evolutionary game model steady state analysis.

Balance Point	Determinant	Trace	Results	Evolutionary Diagram
$E_{1}\left(0,0\right)$	+	−	ESS	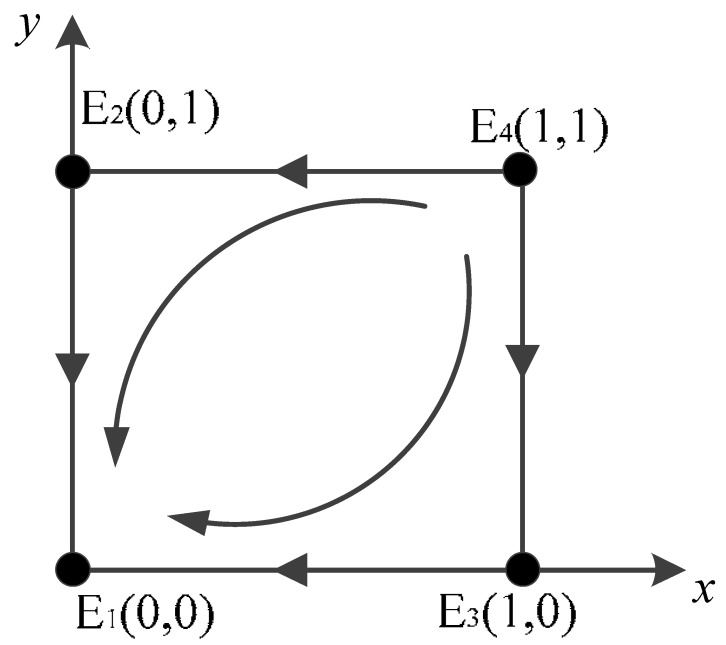
$E_{2}\left(0,1\right)$	−	±	Saddle Point	
$E_{3}\left(1,0\right)$	−	±	Saddle Point	
$E_{4}\left(1,1\right)$	+	+	Instability point	

**Table 5:** Scenario 2 evolutionary game model steady state analysis.

Balance Point	Determinant	Trace	Results	Evolutionary Diagram
$E_{1}\left(0,0\right)$	−	±	Saddle Point	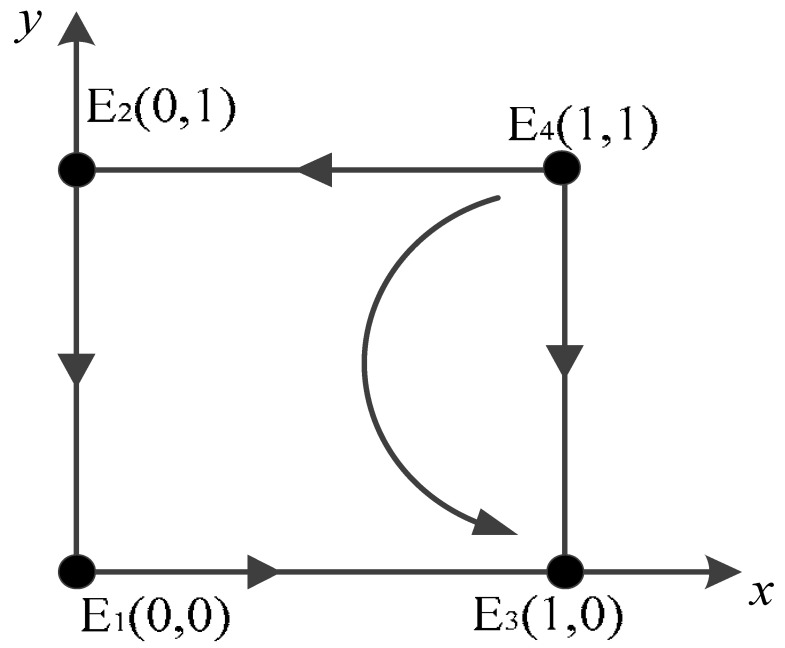
$E_{2}\left(0,1\right)$	−	±	Saddle Point	
$E_{3}\left(1,0\right)$	+	−	ESS	
$E_{4}\left(1,1\right)$	+	+	Instability point	

**Table 6:** Scenario 3 evolutionary game model steady state analysis.

Balance Point	Determinant	Trace	Results	Evolutionary Diagram
$E_{1}\left(0,0\right)$	+	−	ESS	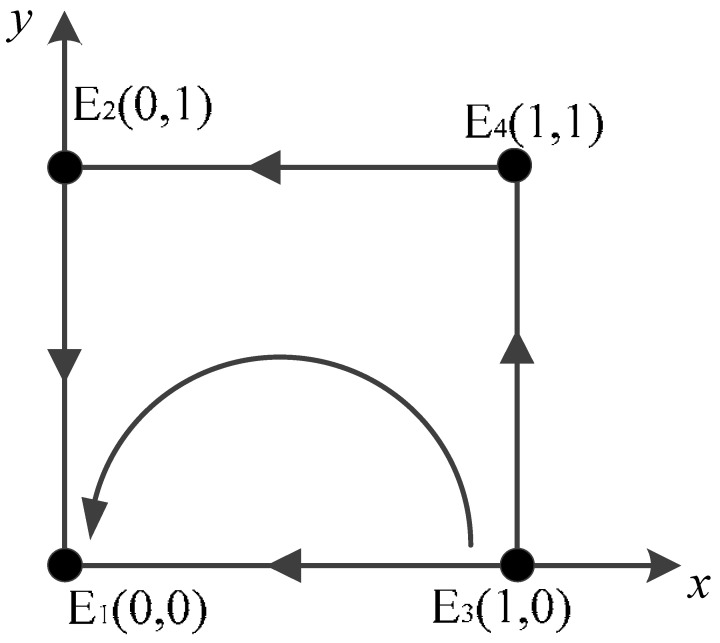
$E_{2}\left(0,1\right)$	−	±	Saddle Point	
$E_{3}\left(1,0\right)$	+	+	Instability point	
$E_{4}\left(1,1\right)$	−	±	Saddle Point	

**Table 7:** Scenario 4: evolutionary game model steady state analysis.

Balance Point	Determinant	Trace	Results	Evolutionary Diagram
$E_{1}\left(0,0\right)$	−	±	Saddle Point	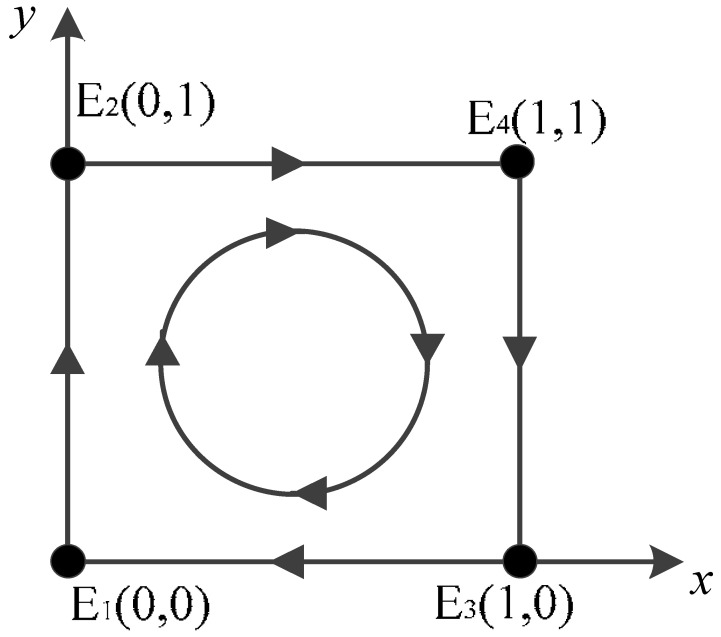
$E_{2}\left(0,1\right)$	−	±	Saddle Point	
$E_{3}\left(1,0\right)$	−	±	Saddle Point	
$E_{4}\left(1,1\right)$	−	±	Saddle Point	

## Data Availability

Not applicable.
